# eHealth Literacy: Knowledge, Attitudes, and Behaviors Among Italian Adolescents

**DOI:** 10.3390/healthcare13222827

**Published:** 2025-11-07

**Authors:** Maria Catone, Giorgia Della Polla

**Affiliations:** Department of Experimental Medicine, University of Campania “Luigi Vanvitelli”, Via Luciano Armanni 5, 80138 Naples, Italy; maria.catone@unicampania.it

**Keywords:** adolescents, eHealth literacy, Internet, Italy, survey

## Abstract

**Background:** eHealth literacy (eHL) is the ability to seek, find, understand, and apply digital health information. Adolescents often overestimate their eHL skills, potentially leading to poor health decisions and posing a public health concern. **Objectives:** This study aimed to examine the knowledge, attitudes, and behaviors toward eHL and seeking health information online among a sample of Italian adolescents aged 10–19 to identify the sociodemographic characteristics and other variables that are associated with the outcomes of interest. **Methods:** A cross-sectional study was conducted using a structured questionnaire, including the eHealth Literacy Scale (eHEALS). **Results:** A total of 793 Italian adolescents from seven public schools in Naples, southern Italy, participated in the survey. Among the participants, 58.6% had an eHEALS score below 27. A higher eHL was associated with being male, perceiving the Internet as useful for health decisions, the use of electronic devices for seeking health information, consulting institutional/scientific sources, having a mother with lower education, and having parents without underlying chronic conditions. Additionally, a higher eHL, male sex, younger age, and Internet use for seeking health information in the last three months were linked to perceiving the Internet as a helpful tool for health decision making. **Conclusions:** Overall, Italian adolescents reported suboptimal eHL with sex, attitudes, and parental factors playing significant roles. Targeted educational interventions are needed to enhance eHL in this specific age group.

## 1. Introduction

The Internet represents the main source of health information [1], but unreliable web-based sources may induce users to adopt harmful behaviors [2]. Thus, it is necessary to acquire the ability to manage online health information and make safe health decisions. eHealth Literacy (eHL), defined as “the ability to search, find, understand, and evaluate health information from electronic sources and to apply the acquired knowledge to address or solve a health problem” [3], is attracting attention from the scientific community due to its role as both a risk and a protective factor that impacts various health-related outcomes [4]. Numerous initiatives have explored this topic across different populations, including college students [5], diverse groups of patients [6,7], and adults [8]. However, a particular emphasis should be placed on adolescents who represent the most digitally connected generation [9]. In Europe, 96% of individuals aged 16–29 use the Internet [10], and adolescents aged 11–19 spend more than five hours per day online [11]. While social media for recreational purposes constitutes a significant portion of their Internet use [12], adolescents also turn to the Internet to seek health information [13]. Worldwide, adolescents exhibit moderate to high eHL [14,15], while in Europe, good eHL levels have been reported [16,17,18]. However, a discrepancy between their self-assessed proficiency and their actual eHL exists [19,20]. eHL represents a determinant that impacts the advantages derived from online health information access [16], making it essential to explore its levels among adolescents. Currently, to the best of our knowledge, there is a lack of scientific evidence on this topic in Italy. Italian data mainly focus on health literacy [21], highlighting the need to explore this specific skill in an understudied European context. Italy represents a relevant case study given that Internet use is pervasive among adolescents [11], reflecting the broader European trend in digital engagement among young populations [22,23]. As European countries share similar challenges regarding adolescents’ Internet use, findings from Italy may provide valuable insights, especially given the increasing reliance on digital platforms for health information among adolescents continent-wide [24].

This cross-sectional study aims to explore the knowledge, attitudes, and behaviors toward eHL and seeking health information online among a sample of Italian adolescents aged 10–19 to identify the sociodemographic characteristics and other variables that are associated with the outcomes of interest. Based on existing evidence and patterns observed in similar populations, we hypothesize that (1) Italian adolescents will demonstrate moderate to good eHL levels and (2) eHL levels will be associated with specific knowledge, attitudes, and/or behaviors related to Internet use for health information-seeking and/or sociodemographic variables.

## 2. Materials and Methods

### 2.1. Setting and Population

This cross-sectional study was conducted from January to March 2025 in 7 public schools (2 middle schools and 5 high schools) located in the Metropolitan Area of Naples, southern Italy. We adopted a two-stage cluster sampling approach: first, public schools were randomly selected from the official list of schools in the Metropolitan Area of Naples (172 in total); second, classrooms were randomly selected within each sampled school, and all the students present on the day of data collection were invited to participate. In Italy, “middle school” refers to lower-secondary education (typically ages 10–13) and “high school” refers to upper-secondary education (typically ages 14–19). Public schools are state-funded institutions. Private schools were not included in the sampling process. To be included, participants had to be 10–19 years old, be physically and cognitively fitted to complete the questionnaire and be authorized by at least one parent/guardian to participate in the study. The required minimum sample size was calculated to be 538, which is based on the assumption that 70% of adolescents would be identified as having sufficient levels of eHL [16,25]. This calculation was made using a 95% confidence level, a 5% margin of error and an expected response rate of 60%.

### 2.2. Study Procedures

The research protocol and questionnaire were approved by the Ethics Committee Campania 2 (protocol number 0032325/i) on 18 December 2024. Prior to data collection, school principals received an invitation letter describing the study objectives and procedures. Once authorization was obtained, data were collected using two different procedures depending on school grade. For high school students, trained investigators distributed paper-based, self-administered questionnaires in the classrooms and supervised their completion. Minors who were interested in participating received consent forms for parental approval as well as assent forms for themselves. In middle schools, the same investigators explained the study aims and distributed sealed envelopes containing an invitation letter, consent and assent forms, and the questionnaire. Students were instructed to complete the questionnaire at home and to return the sealed envelope to the school on agreed collection dates. For participants <18 years, participation required both written parental/guardian consent and adolescent assent. For participants aged 18–19 years, written informed consent was obtained directly from the adolescent. Students who did not provide assent or informed consent did not receive the questionnaire. Participants were informed about voluntary participation, anonymity, confidentiality, and the scientific purpose of the study. Five follow-up efforts were coordinated with teachers to maximize response rates. No incentives were offered.

### 2.3. Questionnaire

The questionnaire was developed based on prior research assessing eHL among adolescents and their use of the Internet to seek health information [26,27,28,29]. A total of 15 participants assessed the consistency and wording of the questionnaire in the pilot phase, and the results were not included in the analysis. The questionnaire consisted of four sections and required approximately 30 min to complete. The first section collected sociodemographic and anamnestic characteristics data on both adolescents (i.e., age, sex, nationality, school grade, having cohabitants, history of chronic medical conditions, medication, self-perceived health status) and their parents (educational level, employment, history of chronic medical conditions). Finally, self-perceived health status was measured on a 10-point Likert-type scale (from 1 = “very poor” to 10 = “excellent”). The second section assessed participants’ knowledge of eHL by asking if they had ever heard about it and the response options were “yes” or “no”, which were followed by a list of options (i.e., personal networks such as relatives/friends, the Internet, social media, healthcare workers, TV/newspapers/radio, institutions such as Ministry of Health, school or other) in case of “yes”. This variable was designed to assess participants’ familiarity with the term of eHL itself and was not related to their eHL levels. The third section examined participants’ eHL levels as well as their attitudes and behaviors toward Internet use to seek health information. The eHealth Literacy Scale (eHEALS) [30] was used to measure eHL. This eight-item tool was originally validated in adolescents and has been translated into Italian [31]. Items are rated on a five-point Likert-type scale ranging from 1 (strongly disagree) to 5 (strongly agree), yielding total scores from 8 to 40 and with higher scores indicating greater self-perceived eHL. The internal consistency of the eHEALS in this study was demonstrated with a Cronbach’s alpha of 0.80, indicating good reliability. Two additional items from the eHEALS, which were not included in the overall eHL score, were used to assess participants’ attitudes toward Internet use to seek health information. These items measured how useful they felt the Internet was in helping them in making decisions about their health and how important it was for them to be able to access health resources on the Internet using a five-point Likert-type scale, ranging from 1 (not useful/not important at all) to 5 (very useful/very important). Participants’ behaviors concerning their online health information seeking were assessed by asking whether they used the Internet with “yes” or “no” responses and for how many hours per day (i.e., less than 1 h/1–2 h/2–3 h/3–4 h/more than 4 h). Additionally, participants were asked if they have used the Internet to seek health information in the past three months with “yes” or “no” and to select the reasons for doing or not doing so from a list of seven reasons in case of “yes” and eight reasons in case of “no”. The fourth section investigated participants’ used sources and health information needs by asking which electronic devices they used to look for health information online (none/computer/tablet/mobile phone/other), which were their health information needs in the last three months by selecting from a list of health topics, the health information sources they usually referred to (search engines/social media/Wikipedia/Institutional websites or scientific journals/traditional media such as TV/newspaper/radio/none/other), and whether they required further information on eHL by answering with “yes” or “no” options.

### 2.4. Statistical Analysis

Statistical analyses were conducted using STATA 19 (College Station, TX, USA) [32]. First, to describe the sample characteristics, knowledge, attitudes, and behaviors measures such as the frequency, mean, range, and standard deviation were used. Distributional assumptions were checked using skewness, kurtosis, and Shapiro-Wilk tests. Second, univariate analysis using a chi-square test or Student’s t-test was performed to assess the associations between categorical and continuous variables and the outcome of interest, respectively. Third, variables with *p* ≤ 0.25 in the univariable analyses were considered for inclusion into the multivariable logistic regression. To preserve an adequate number of observations and statistical power, not all eligible variables were ultimately included in the following step. We then fitted two binary logistic regression models, each with a different dependent variable, to identify independent associations: Model 1 predicted sufficient eHealth literacy depending on eHEALS scores, which were dichotomized into limited (8–26 points) and sufficient (27–40 points) eHL levels [33]; Model 2 predicted perceiving the Internet as useful for health decision making (Likert < 4 vs. ≥4). The following independent variables have been tested for the outcomes: age in years (continuous), sex (male = 0; female = 1), nationality (other = 0; Italian = 1), school grade (middle school = 0; high school = 1), having cohabitants (continuous), mother’s educational level (high school degree or less = 0; baccalaureate/graduate degree = 1), father’s educational level (high school degree or less = 0; baccalaureate/graduate degree = 1), mother being healthcare worker (no = 0; yes = 1), father being healthcare worker (no = 0; yes = 1), having a chronic medical condition (no = 0; yes = 1), taking at least one medication (no = 0; yes = 1), having parents with underlying chronic medical conditions (no = 0; yes = 1), self-perceived health status (continuous), having heard about eHL (no = 0; yes = 1), having sufficient eHL levels (eHEALS score < 27 = 0; eHEALS score ≥ 27 = 1); having heard about eHL by healthcare workers or institutions such as the Ministry of Health (no = 0; yes = 1), utility of the Internet for health decision making (scoring < 4 = 0; scoring ≥ 4 = 1), importance of being able to access health resources on the Internet (scoring < 4 = 0; scoring ≥ 4 = 1), hours per day spent on the Internet (1 to 3 h = 0/from 3 to more than 4 h = 1), use of the Internet to seek health information in the last three months (no = 0; yes = 1), believing to retrieve with ease health information from the Internet (no = 0; yes = 1), believing that online health information is reliable (no = 0; yes = 1), having used electronic devices to seek health information on the Internet (no = 0; yes = 1), having had at least one health information need in the last three months (no = 0; yes = 1), having used institutional websites or scientific journals to look for health information (no = 0; yes = 1), and need of additional information about eHL (no = 0; yes = 1). The values of *p* = 0.2 and *p* = 0.4 were used to select candidate variables for retention and exclusion in the final multivariable binary logistic regression models. Results of the logistic regression models were measured using adjusted odds ratios (aOR) with 95% confidence intervals (CIs). For all analyses, two-tailed tests were used, and *p*-values ≤ 0.05 were considered statistically significant. Cases with missing data for the variables included in the analyses were excluded listwise. No data imputation was performed.

## 3. Results

A total of 793 questionnaires were returned with a response rate of 68.5% of the 1157 students approached. According to recent demographic data [34], the population aged 10–19 years in the Province of Naples amounts to 325,320 individuals. Given this reference population, the final sample ensures adequate representativeness, corresponding to an estimated margin of error of approximately ± 3.5 percentage points at a 95% confidence level under simple random sampling assumptions. The main characteristics of the respondents are summarized in Table 1.

### 3.1. Adolescents’ Knowledge of eHealth Literacy

For survey items on participants’ knowledge, most of the participants (86.2%) reported they had never heard about eHL. Among those who did, the reported sources of information about eHL were the Internet (43.1%), social media (29.4%), school (24.8%), and relatives/friends (21.1%). Healthcare workers (11.1%), and TV/newspapers/radio played a lesser role in disseminating information about eHL (17.4%) with institutions such as the Ministry of Health having the lowest reach (10.1%).

### 3.2. Adolescents’ Attitudes and Online Health Information Seeking Behaviors

For the items measuring attitudes (5-point scales), 21.3% of participants scored ≥4 for the usefulness of the Internet in health decision making, while 30.5% scored ≥4 for the importance of being able to access health resources online. Overall, 99.9% of the sample reported using the Internet with the majority spending more than 3 h per day (62.2%) and, in the last three months, more than half of participants (59.7%) used the Internet to seek health information. The main reasons for consulting online sources for health information were personal interest (73.9%), ease of access to health information (44.2%), the belief that they knew reliable health information sources (16.2%), the belief that the health information retrieved online was reliable (15.5%), the need to guarantee personal privacy (11.9%), and the inability to consult medical doctors (5.6%). Among the participants who did not search for health information online in the last three months (40.3%), 56.7% did not have the ability to seek health information, 46.4% believed that web-based health information is not reliable, and 21.7% did not believe they knew reliable health information sources. Furthermore, 8.6% and 8%, respectively, believed that it was difficult to identify reliable health information among the search results and to understand the used language, while 4.8% had difficulty exploring websites to retrieve health information.

### 3.3. Adolescents’ eHealth Literacy Levels

Overall, 58.6% of participants had an eHEALS score below 27. Data distribution was approximately symmetric (skewness = −0.40; kurtosis = 3.31; Shapiro-Wilk W = 0.989, *p* < 0.001, n = 751). The items “I know how to find helpful health resources on the Internet” and “I can tell high-quality health resources from low-quality health resources on the Internet” had the highest scores with mean values of 3.3 ± 0.9 and 3.3 ± 1.1, respectively. The items “I feel confident in using information from the Internet to make health decisions” and “I know where to find helpful health resources on the Internet” had the lowest scores with mean values of 2.7 ± 1.2. and 2.9 ± 1, respectively.

After the univariate analysis (Table 2), two multivariate logistic regression models were fitted to assess which factors were more likely to affect the outcomes of interest (Table 3). Adolescents who were male (aOR = 0.58; 95% CI = 0.41–0.82), those who believed that the Internet is useful for health decision making (aOR = 1.95; 95% CI = 1.22–3.12), those who used electronic devices to look for health information online (aOR = 3.42; 95% CI = 1.33–8.77), those who used institutional websites or scientific journals as source of information (aOR = 1.62; 95% CI = 1.10–2.39), those who had a mother with a high school degree or less (aOR = 0.61; 95% CI = 0.40–0.92), and those who had parents without underlying chronic medical conditions (aOR = 0.57; 95% CI = 0.33–0.99) were more likely to have sufficient eHL levels (Model 1). Additionally, Model 2 showed that adolescents who had sufficient eHL (aOR = 2.31; 95% CI = 1.56–3.43), who were male (aOR = 0.45; 95% CI = 0.30–0.68), young (aOR = 0.79; 95% CI = 0.71–0.87), and who used the Internet to seek health information in the last three months (aOR = 2.79; 95% CI = 1.70–4.59) were more likely to believe that the Internet is useful for health decision making (Model 2 in Table 3).

### 3.4. Adolescents’ Health Information Tools, Needs and Sources

Most of the samples reported using electronic devices to look for health information (87%), among which the mobile phone was the most used (97%), which was followed by computers (14.8%) and tablets (6.1%). When asked about health information needs in the last three months, the most common topics were nutrition (44.2%), skin problems such as acne (30.4%), anxiety (29.7%), physical activity (26.9%), sexual intercourse (24.4%), menstruation (22.6%), and infectious diseases (19.8%). A total of 91.1% of participants reported using at least one information source, where search engines (86.4%) and social media (34.4%) were the most prevalent, which was followed by institutional websites and scientific journals (28%), Wikipedia (21.8%), and traditional media (TV/newspapers/radio) (8.7%). Finally, almost half of the sample (45.6%) expressed the need for more information on eHL.

## 4. Discussion

This cross-sectional survey aimed to provide new insights into adolescents’ eHL levels and their knowledge, attitudes, and behaviors toward this topic and the use of the Internet to seek health information. Despite frequent and daily Internet use among adolescents, most participants reported suboptimal eHL levels. Our findings highlight several key factors associated with both eHL and the belief of the Internet as useful for health decision making.

Sufficient eHL was associated with being male, perceiving the Internet as useful for health decision making, using electronic devices to search for health information, consulting institutional/scientific sources, lower mother’s education level and an absence of parental chronic medical conditions. These adjusted associations highlight that digital engagement, positive attitudes, and reliance on credible sources are linked to higher eHL after accounting for sociodemographic and behavioral covariates.

First, our findings show significant sex differences in eHL with male adolescents reporting higher eHEALS scores than females and being more likely to perceive the Internet as useful for health decision making. While previous research generally suggests that sex does not significantly influence adolescents’ eHL [25,35], recent studies align with our findings [36,37]. For example, the study by Terzi et al. [37] conducted among adolescents aged 14–17 years showed that males had higher eHL. One possible reason is that males may perceive greater confidence in their digital literacy skills, which may contribute to higher self-perceived eHL scores [38,39]. A recent meta-analysis indicated that despite a narrowing gender gap in technology use, males tend to have a more positive attitude toward technology than females [38]. Traditional masculine norms, such as self-reliance and emotional restraint, may also influence male adolescents’ health-seeking behavior, leading them to prefer digital resources rather than seeking direct social or professional support [36,40]. Moreover, male adolescents are less inclined than females to seek support from their social networks or health professionals even in situations of significant distress and preferring self-help strategies [41]. Therefore, targeted educational interventions are needed to enhance eHL levels among adolescents and eventually address underlying gender disparities.

The improvement of adolescents’ eHL could directly impact their overall health awareness and health-seeking behaviors, as higher eHL fosters the identification of credible online information and engagement in preventive behaviors [8]. In the Italian context, where digital health information is easily accessible, the enhancement of eHL may represent an essential strategy to promote a conscious and autonomous approach to health among young people.

Schools, as key settings for adolescents’ behavior shaping, play a pivotal role in fostering their health literacy competencies [40,42]. Given the limited research on gender-specific trajectories in eHL development during adolescence, further investigation is warranted to substantiate these observations and explore broader implications.

Regarding attitudes, our results reveal several predictors of adolescents’ belief that the Internet is useful for health decision making, which may shape related behaviors.

In fact, adolescents believing that the Internet is useful for health decision making reported having higher eHL levels and vice versa, indicating a positive association consistent with prior research [14,43]. Hernández-Rabanal et al. [43] demonstrated that adolescents aged 15–18 considered the Internet important to access health resources and, following an intervention, they were also more likely to believe that the Internet is useful for health decision making. Adolescents who perceive the Internet as useful for making health decisions may be more likely to use it, which in turn may increase the exposure, practice, and refinement of their digital health skills. Therefore, by highlighting the importance and utility of emerging technologies, we may foster skill development among adolescents while reinforcing that information skills extend beyond functional know-how and involve critical competencies [43].

Moreover, we found that adolescents who used the Internet to seek health information in the last three months had a better attitude toward Internet usefulness for health decision making. Our findings suggest that attitudes toward the Internet serve as a psychological bridge between health consciousness and health-related Internet use. This is consistent with the Theory of Planned Behavior [44], which states that attitudes toward a behavior shape behavioral intentions and engagement. However, although positive attitudes facilitate engagement, they do not necessarily ensure competence in evaluating online health information. This highlights the need for interventions aimed at enhancing critical digital literacy [19,20]. Taken together, these findings suggest that both higher eHL and recent, active engagement with online health information are linked to more favorable appraisals of the Internet’s usefulness for health decisions beyond sociodemographic and behavioral confounders.

Additional findings include the influence of parental education and health status on adolescents’ eHL. Surprisingly, participants whose mothers had a high school degree or less exhibited higher eHL levels, which contrasts with prior studies suggesting that higher parental education is typically associated with better health literacy [25,35]. Adolescents are expected to ask their family for health-related information. However, when their health inquiries surpass their parents’ knowledge, they may increasingly rely on the Internet to find explanations, fostering greater independence in navigating digital health resources [45]. Therefore, a possible explanation for this unexpected finding is that adolescents with less educated mothers may develop greater digital autonomy in seeking health information online due to reduced parental guidance. Nevertheless, further research is needed to confirm this association.

Age appears to have a negative relationship with digital health literacy with younger individuals demonstrating higher proficiency in digital navigation and online health information-seeking behaviors [23]. The results of this survey further support this trend, revealing that the use of electronic devices for seeking health information is positively associated with eHL. Moreover, when looking at the attitudes, younger individuals were more likely to believe that the Internet is useful for health-related purposes, suggesting that confidence in digital devices enhances the ability to access and use health-related information [46]. Since technology fluency is a core component of eHL [3], digital education initiatives tailored to both adolescents and their parents are needed.

Moreover, adolescents whose parents do not have underlying chronic medical conditions reported higher levels of eHL. We hypothesize that adolescents with limited exposure to healthcare services due to their parents’ good health may frequently rely on digital resources for health-related information. Unlike individuals who frequently interact with healthcare professionals who may directly answer to their health concerns, these adolescents might develop stronger eHL to independently seek, assess, and interpret health information online [47]. Previous research has shown that adolescents usually prefer to consult online resources to address their health information needs [40], which are the most used in our sample.

In particular, we found a positive association between the use of institutional websites or scientific journals and eHL. This finding is consistent with prior research, indicating that familiarity with reliable health resources bolsters adolescents’ online health information-seeking efficacy [15,48]. However, only a minority of the sample reported using institutional websites or scientific journals, highlighting an urgent need for educational interventions promoting awareness regarding reliable health information sources. Indeed, after search engines, social media were highly used by our sample to seek health information, which is consistent with prior research [24]. Although adolescents generally distrust health information retrieved from social media, they continue to use these sources due to their convenience, easy accessibility, familiarity, and relevance while engaging in other social media activities [20,24]. This suggests that adolescents’ health information-seeking behavior is influenced by both the accessibility and trustworthiness of sources. Therefore, efforts should focus on improving adolescents’ ability to identify and use high-quality resources [3].

These findings have significant implications for health education and adolescent well-being. For this reason, the World Health Organization (WHO) has emphasized the importance of addressing health literacy at the school level through international and regional policy frameworks [49]. Research is needed to develop interventions aimed at improving eHL among adolescents. For example, structured training sessions in high schools may enhance students’ attitudes toward online health information seeking and their eHL [43]. Additionally, involving adolescents in the co-design of such programs may further increase their engagement and motivation. Parents, educators, and healthcare workers should play an active role in guiding them toward credible online health resources while promoting eHL as a lifelong skill. Furthermore, health literacy initiatives should be adapted to diverse populations and promoted at the institutional level, as recommended by the WHO [49]. Finally, the development of youth-friendly, easy-to-understand, and visually appealing online health platforms is essential to ensure adolescents’ access to reliable web-based health information [24].

This study presents some limitations that should be considered when interpreting results. The cross-sectional design precludes the establishment of causal relationships, and self-reported data may be subject to recall or social desirability biases. Missing data were excluded listwise, which might have reduced the effective sample size for some analyses, although the overall number of participants remained large. Furthermore, the sample was limited to the metropolitan area of Naples, which may restrict generalizability to rural Italian populations, private school students, or adolescents from different socioeconomic contexts across Italy and other European countries. Future research should adopt longitudinal designs including different geographic and socioeconomic contexts. Moreover, some findings may have benefited from the assessment of other variables that were not examined in this study, such as parents’ eHL, their use of the Internet to seek health information, and socioeconomic status. The sample included fewer middle- than high-school students, which may limit the precision and generalizability of results to middle-school populations. Finally, eHealth literacy was assessed using the eHEALS scale, which measures self-perceived competency rather than objective performance, limiting our understanding of their actual competencies. There are several strengths of the present survey. To the best of our knowledge, this is the first study to investigate eHL among Italian adolescents aged 10–19 years, addressing a critical knowledge gap in European adolescent digital health research and providing a basis for future research and policy development. The diverse sample included equal sex distribution, enabling gender-specific analyses that revealed important disparities requiring tailored educational approaches. In addition, by identifying the most common health information needs, the predictors of eHL and attitudes toward the Internet as useful for health decision making, this research highlights priority areas where collaborative efforts between schools and healthcare professionals could be most effective. Moreover, the timing of our assessment in the post-COVID era captured contemporary digital behaviors when online health information seeking has substantially increased among young people.

## 5. Conclusions

In conclusion, we observed suboptimal eHL levels among Italian adolescents, highlighting the need for targeted interventions. Sufficient eHL was associated with being male, considering the Internet useful for making health-related decisions, using electronic devices to seek health information, consulting institutional or scientific sources, having a mother with a lower education level, and having parents without chronic medical condition. Moreover, greater eHL, being male, younger age, and recent (past three months) use of the Internet for health information were associated with perceiving the Internet as useful in health decision making. Key areas should include eHL training, recognizing gender disparities, parental influences, and promoting the use of reliable health sources. Schools and policymakers should enhance multidisciplinary approaches involving educators and healthcare professionals to enhance adolescents’ knowledge, attitudes, and behaviors toward Internet use for health-related purposes. Future research should explore long-term eHL development and effective intervention strategies.

## Figures and Tables

**Table 1:** Sociodemographic and anamnestic information of participants.

Adolescents’ Characteristics	N	Mean (±SD)	%
Age, in years (10–19)		15.8 (±2.2)	
Middle school (10–13)		11.9 (±0.9)	14.9
High school (14–19)		16.5 (±1.5)	85.1
Sex			
Male	393		50
Female	393		50
Nationality			
Italian	779		98.2
Other	14		1.8
School grade			
Middle school	118		14.9
High school	675		85.1
Having cohabitants			
≤2	129	3.3 (±1.0)	16.5
≥3	651		83.5
Chronic medical condition *			
Yes	75		9.7
No	700		90.3
Medication *			
Yes	61		81.3
No	14		18.7
Self-perceived health status		8.5 (±1.3)	
**Parents’ characteristics**	**N**	**Mean (**±**SD)**	**%**
Mother’s educational level			
High school degree or less	577		74.2
Baccalaureate/graduate degree	201		25.8
Father’s educational level			
High school degree or less	627		82.7
Baccalaureate/graduate degree	131		17.3
Mother’s professional status			
Employed	388		53.2
Unemployed	341		46.8
Father’s professional status			
Employed	718		97.2
Unemployed	21		2.8
Mother being a healthcare worker			
Yes	45		6.2
No	684		93.8
Father being a healthcare worker			
Yes	32		4.3
No	707		95.7
Parent with underlying chronic medical condition *			
Yes	92		11.8
No	685		88.2

Number for each item may not add up to the total number of the study population due to missing values; * more than one option was allowed to be indicated.

**Table 2:** Results of the univariate analysis.

Independent Variables	Model 1 eHEALS Score ≥27 (41.4%)	Model 2 Believing That Internet Is Useful for Health Decision Making (Likert Score ≥ 4) (21.3%)
Age in years (continuous)	0.3786	0.0203
Sex	<0.001	<0.001
Nationality	0.359	0.185
School grade	0.065	0.392
Having cohabitants (continuous)	0.6957	0.5751
Mother’s educational level	0.017	0.740
Father’s educational level	0.115	0.525
Mother being a healthcare worker	0.697	0.620
Father being a healthcare worker	0.656	0.892
Having a chronic medical condition	0.951	0.141
Taking at least one medication	0.683	0.428
Having parents with underlying chronic medical conditions	0.067	0.492
Self-perceived health status (continuous)	0.9608	0.7498
Having heard about eHL	0.052	0.646
Having heard about eHL by healthcare workers or institutions such as Ministry of Health	0.464	0.936
Having sufficient eHL levels	-	<0.001
Utility of the Internet for health decision making	<0.001	-
Importance of being able to access health resources on the Internet	<0.001	<0.001
Hours per day spent on the Internet	0.780	0.556
Use of the Internet to seek health information in the last three months	<0.001	<0.001
Believing one can easily retrieve health information from the web	0.002	0.187
Believing that online health information is reliable	0.027	<0.001
Having used electronic devices to seek health information on the Internet	<0.001	0.001
Having had at least one health information need in the last three months	0.009	0.003
Having used institutional websites or scientific journals to look for health information	<0.001	0.468
Need for additional information about eHL	0.407	0.723

Model 1 outcome = sufficient eHL (eHEALS ≥ 27); Model 2 outcome = Believing that the Internet is useful for health decision making (≥4). Chi-square tests were used for categorical variables and *t*-tests were used for continuous variables. Reported values are *p*-values from univariate analyses. “-“ indicates the outcome variable that has not been analyzed as a predictor in that model.

**Table 3:** Results of the multivariate logistic regression analysis showing the predictors of the outcomes of interest.

Variable	aOR	SE	95% CI	*p*
Model 1. Having sufficient eHL levels (eHEALS score ≥ 27) Log likelihood = −375.73, χ^2^ = 61.20 (10 df), *p* < 0.0001 Pseudo R^2^ = 0.0753 Nagelkerke R^2^ ≈ 0.13				
Male	0.58	0.10	0.41–0.82	0.002
Believing that the Internet is useful for health decision making	1.95	0.47	1.22–3.12	0.005
Having used electronic devices for health information seeking	3.42	1.64	1.33–8.77	0.011
Having used institutional websites or scientific journals as source of information	1.62	0.32	1.10–2.39	0.014
Having a mother with high school degree or less	0.61	0.13	0.40–0.92	0.017
Having parents without underlying chronic medical condition	0.57	0.16	0.33–0.99	0.044
Having heard about eHL	1.40	0.34	0.86–2.26	0.177
Believing that being able to access health resources on the Internet is important	1.31	0.27	0.87–1.98	0.191
Attending middle school	0.76	0.21	0.44–1.30	0.314
Having used the Internet to seek health information in the last three months	1.20	0.23	0.82–1.76	0.341
Model 2. Believing that the Internet is useful for health decision making (Likert score ≥ 4) Log likelihood = −320.16, χ^2^ = 82.57 (5 df), *p* < 0.0001 Pseudo R^2^ = 0.1142 Nagelkerke R^2^ ≈ 0.19				
Having sufficient eHealth literacy levels	2.31	0.47	1.56–3.43	<0.001
Male	0.45	0.09	0.30–0.68	<0.001
Young	0.79	0.04	0.71–0.87	<0.001
Having used the Internet to seek health information in the last three months	2.79	0.71	1.70–4.59	<0.001
Having needed more information about a health topic in the last three months	1.73	0.54	0.94–3.20	0.078

## Data Availability

The anonymous data presented in this study are available on request from the corresponding author. Access to these data is restricted in order to safeguard the privacy and confidentiality of the study participants, in accordance with the informed consent terms.

## Abbreviations

The following abbreviations are used in this manuscript:

eHL	eHealth Literacy
eHEALS	eHealth Literacy Scale
WHO	World Health Organization
SD	Standard Deviation
aOR	Adjusted Odds Ratio
SE	Standard Error
CI	Confidence Intervals
